# Natural Oral Absorption of Therapeutic Peptides: Mechanisms and Physiological Determinants

**DOI:** 10.3390/pharmaceutics18091091

**Published:** 2026-08-29

**Authors:** Lania Ali Hussein, Alexandra Nedic, Andrejs Sitovs, Valentyn Mohylyuk

**Affiliations:** 1 Faculty of Medicine, Riga Stradins University, LV-1007 Riga, Latvia; 052504@rsu.edu.lv (L.A.H.); 051665@rsu.edu.lv (A.N.); 2 Leading Research Group, Faculty of Pharmacy, Riga Stradins University, LV-1007 Riga, Latvia

**Keywords:** peptide drugs, oral bioavailability, permeation, drug transport

## Abstract

Although oral peptide drug administration is the most desirable for both patients and healthcare systems, it is a major pharmaceutical challenge. Typically, less than 1% of orally administered peptide therapeutics reaches the systemic circulation. Modern research has focused on overcoming the physiological barriers responsible for the poor peptide drug bioavailability, while little attention has been given to understanding how measurable absorption occurs despite the barriers. This review aimed to explore the physiological factors that influence oral peptide absorption, such as enzymatic degradation and pH effects, motility, microbiota, transporters, tight junctions, and the effect of inflammation. The available data indicate that these factors function not only as barriers but may also create limited opportunities for peptide uptake. Enzymatic degradation and microbial metabolism reduce the availability of intact peptide drugs yet may generate biologically relevant fragments. Tight junctions are generally regarded as restrictive barriers but are shown to exhibit dynamic regulations that may permit transient paracellular transport. Similarly, gastrointestinal motility, microbiota, and inflammatory processes influence epithelial permeability and duration of peptide exposure both to absorptive surfaces and to degradative enzymes. In contrast, transporter-mediated uptake appears unlikely to account for the absorption of intact peptide drugs due to their substrate size limitations. Together, the findings suggest that measurable oral peptide absorption is unlikely to result from a single dominant pathway but rather from the cumulative contribution of the mechanisms. Improved understanding of these endogenous mechanisms may open opportunities to enhance oral peptide bioavailability and support the development of more effective therapeutics.

## 1. Introduction

Therapeutic peptides are biologically active substances composed of short chains of amino acids linked by peptide bonds that help regulate many physiological processes in the body [1,2,3,4]. Therapeutic peptides, as modern pharmaceuticals, are used as synthetic or recombinant analogs of endogenous peptides in many chronic illnesses such as diabetes, obesity, and cancer, typically exhibiting high target specificity and low systemic toxicity [5]. However, the physicochemical properties of peptide drugs pose significant challenges to the formulation and delivery strategies, resulting in parenteral administration routes, particularly via intramuscular, subcutaneous, or intravenous injections, which limit patient compliance. Oral administration of peptide drugs is highly desirable for both patients and healthcare systems, as it could improve patient compliance and comfort, particularly for chronic conditions requiring long-term treatment. Oral administration of peptides may also lower treatment costs, as it will eliminate the need for injection-related healthcare resource utilization, such as sterile management and clinical supervision of the injections, making the treatment more widely available [5,6,7].

Naturally, less than 1% of orally administered peptides reach the systemic circulation intact, which could be due to the physicochemical properties of the drugs but also to the physiological barriers of the human body [8,9,10]. These factors include proteolytic degradation in the stomach and small intestine, variable pH throughout the gastrointestinal tract (GIT), poor epithelial permeability due to, for instance, tight junctions (TJ), and rapid first-pass metabolism [11]. The intestinal epithelial cells are considered one of the primary biological barriers that prevent drugs from reaching their target receptors. Peptide drugs can cross the intestinal epithelium through two principal pathways: the transcellular route, which involves passage through epithelial cells, and the paracellular route, which occurs between adjacent epithelial cells. However, both pathways are highly restrictive under physiological conditions due to the physicochemical properties of the drug. The transcellular pathway allows passage of very small hydrophobic fragments of peptides (such as residues rich in leucine, valine, isoleucine, and phenylalanine) that undergo passive diffusion, or partially digested peptide fragments [5]. One of the reasons is that the lipophilic membrane makes the transcellular permeability of large, hydrophilic molecules difficult [12]. For the paracellular pathway, TJ between cells also poses a challenge for large molecules, as they are reported to have a pore size of approximately 4–8 Å, but could be expanded to 10–30 Å [13]. The molecular weight cutoff for paracellular transport will thereby be approximately slightly above 3–5 kDa [13].

On the contrary, measurable systemic absorption of peptide drugs has been reported even in the absence of permeation enhancers or specialized formulations, suggesting that a limited proportion of peptides may traverse the intestinal barrier [7].

Although oral bioavailability for most therapeutic peptides is frequently reported to be below 1%, this value should not be interpreted as universal [7,10]. Oral peptide absorption exists along a continuum determined by the interplay between intrinsic molecular properties and the physiological barriers of the GIT [7,10]. Molecular weight, amino acid sequence, and conformational flexibility to enzymatic degradation collectively influence permeability, metabolic stability, and the likelihood that an intact peptide reaches the absorptive epithelium. Also, structural modifications such as cyclization, lipidation, PEGylation, etc., can result in marked differences in oral absorption between classes [14,15,16].

Intestinal permeability of peptide drugs is commonly evaluated using in vitro and ex vivo methods, such as Caco-2 cell monolayers and Ussing chambers with excised intestinal tissue, that provide results in standard metrics such as an apparent permeability coefficient (P_app_). The transepithelial electrical resistance (TEER) measures the electrical resistance across a cell monolayer and reflects both its integrity and tight junctions’ tightness. TEER is used to assess the barrier function and monolayer quality [17].

Recent pharmaceutical advances have enabled the oral delivery of selected peptide drugs through formulation-based strategies. Permeation enhancers are used in formulations with oral peptide drugs to increase the intestinal permeability and thereby to improve their bioavailability. They do not directly affect the drug solubility, but can facilitate transport across the gut wall by different strategies, such as reversibly opening TJ, increasing pH, perturbing cell membranes, or forming non-covalent complexes, enhancing peptide drug passage [18]. For instance, the widely studied permeation enhancers are salcaprozate sodium (SNAC), sodium caprate (C10), and bile acids, which are used in recently approved oral peptide formulations, such as oral GLP-1 analogs, administered together with SNAC or C10 [19].

A prominent example is oral semaglutide, a glucagon-like peptide-1 (GLP-1) receptor agonist co-formulated with the absorption enhancer SNAC, one of the first orally administered GLP-1 analogs approved for clinical use today. However, a high dose of permeation enhancer is required to achieve therapeutically relevant systemic exposure [20]. Oral semaglutide, Rybelsus^®^, is clinically approved for the treatment of type 2 diabetes [21], and a more recent approval of another oral semaglutide, Wegovy^®^ [22], for chronic weight management further demonstrates the clinical applicability of oral peptide delivery. Despite promising data, the strategies still yield low and variable bioavailability; for instance, the oral bioavailability of GLP-1 analogs administered together with SNAC are reported to show an oral bioavailability of approximately 0.8–1% in human studies [18,23,24].

There are also formulation approaches, including nanoparticles, self-emulsifying drug delivery systems, hydrogels and enzyme inhibitors, that have been investigated, for example, as coatings on peptide drugs to improve the stability and oral bioavailability of the peptide drugs [25].

Importantly, formulation-assisted strategies, while clinically successful, do not account for the small but measurable fraction of natural oral peptide absorption. The mechanisms underlying this <1% remain incompletely understood, although considerable effort has been devoted to identifying physiological barriers that restrict oral peptide bioavailability; current research has largely prioritized the development of artificial delivery systems rather than exploring the biological processes contributing to the baseline peptide transport across the gastrointestinal epithelium. A mechanistic understanding of these processes is critical for identifying modifiable physiological pathways that may be exploited in future oral peptide drug development. This review addresses that gap by critically examining the current evidence for the mechanism underlying endogenous peptide absorption, as this review aims to explore the current published literature and gather information on the physicochemical and physiological factors that contribute to the unfacilitated measurable oral absorption of peptide drugs, and to identify current knowledge gaps and leverage points that could inform the development of future strategies to improve the oral bioavailability of peptide drugs. To address this objective, six mechanisms that could hypothetically contribute to the oral bioavailability of peptide drugs were explored. Our investigation explored the contributions of enzymatic degradation and pH effects, motility, microbiota, transporters, TJ, and the effect of GIT inflammation.

This hypothesis-driven narrative review was formed by a comprehensive literature search conducted using Google Scholar and PubMed. Searches were performed using combinations of keywords related to natural oral peptide absorption and bioavailability, including oral peptide bioavailability, oral peptide absorption, intestinal permeability, TJ, intestinal inflammation, gut microbiota, proteolytic enzymes, intestinal pH, peptide transporters and additional mechanism-specific terms relevant to natural peptide absorption. The search strategy was structured around the six mechanistic hypotheses discussed in this review: (I) tight junctions, (II) enzymatic degradation and effects of gastrointestinal pH, (III) impact of active transport, (IV) microbiota, (V) inflammation, and (VI) GIT motility. Publications available up to 2026 were considered; both original research articles and review articles published in English were evaluated. Priority was given to original mechanistic studies whenever available, whereas review articles were used to provide scientific context and to identify additional primary studies through manual screening of their reference lists. Due to the limited number of original studies directly investigating the topic, evidence from both primary and secondary literature was critically synthesized. The included literature was selected based on its relevance to the review objectives, scientific credibility, methodological rigor, and contribution to advancing the current understanding.

## 2. Tight Junctions

**Figure 1:**
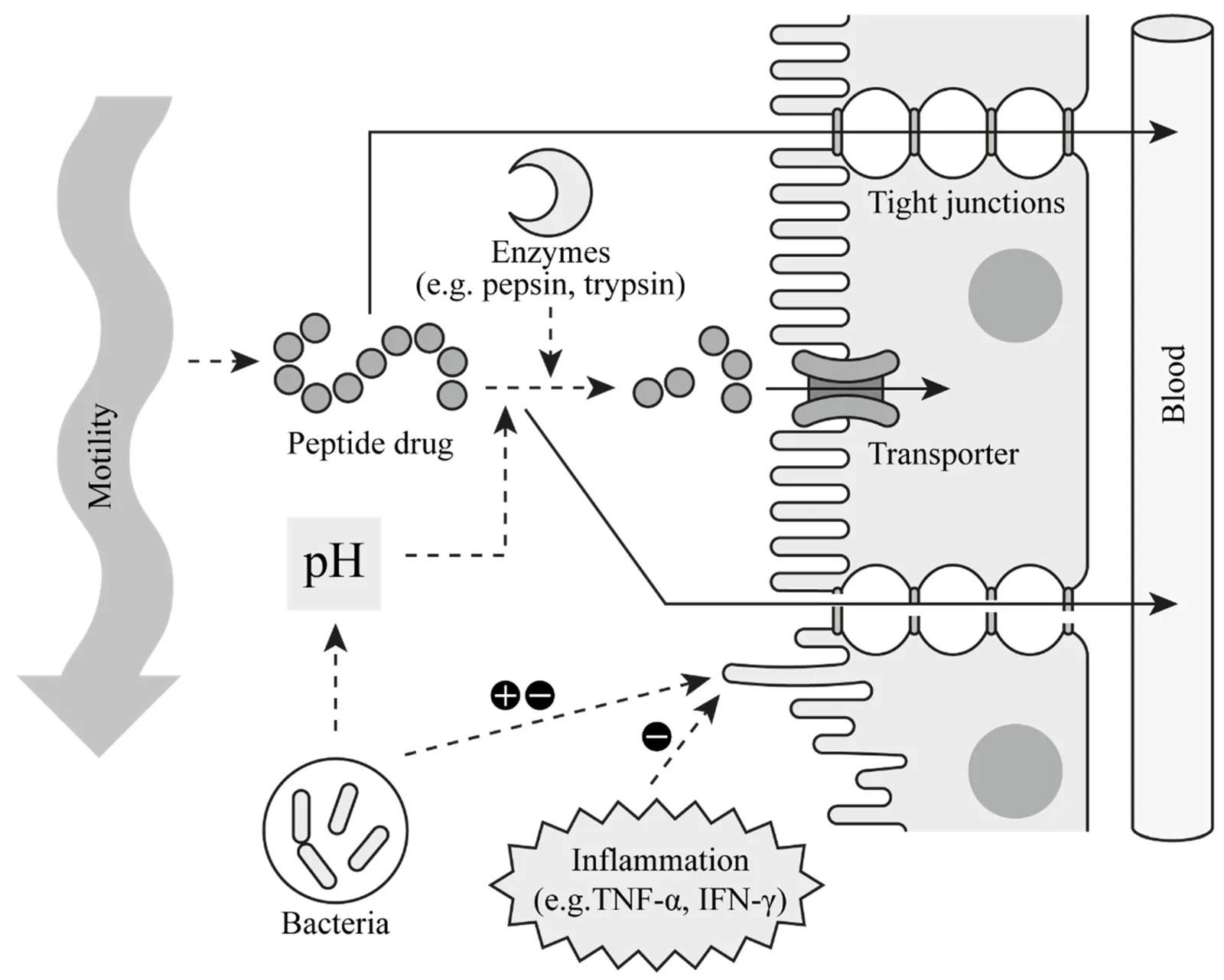
Schematic representation of peptide drug absorption from the GIT. Dashedarrows represent factors affecting peptide absorption. Solidarrows represent peptide transport.

Tight junctions (Figure 1) are zones composed of transmembrane proteins that seal the space between intestinal epithelial cells, creating a selective, semi-permeable barrier that prevents ions and molecules from passing through the paracellular space [18]. This barrier has two pathways: a size- and charge-selective pathway, also called the pore pathway, and a low-capacity leak pathway. Tight junctions comprise proteins such as claudins, occludins, tricellulin, scaffold proteins and junctional adhesion molecules (JAMs), among others.

Claudins form the main sealing strands of TJ, which determine the ion selectivity of the paracellular pathway; occludins contribute to TJ structure and regulation of barrier permeability; tricellulin is located where three cells meet and helps maintain barrier integrity. The junctional adhesion molecules and scaffold proteins connect transmembrane TJ to the cytoskeleton and organize the TJ complex [26,27]. The pore pathway is primarily built upon claudin expression, forming a system of small pores with a radius of approximately 0.4 nm (4 Å) [28]. The claudin family of TJ proteins has several different isoforms, which have different permeability properties. For instance, pore-forming claudins (claudin-2 and 15) allow for paracellular passage of ions and solutes with a molecular weight less than 400 Da, while sealing claudins such as claudin-4, 5, and 8 restrict passage of such molecules. Expression of the TJ proteins varies along the GIT. For instance, colonic crypt bases are reported to have leakier junctions compared to luminal epithelial surfaces. Also, the other TJ proteins allow for some other rare, larger molecule leaks, such as in the case with tricellulin, which seals the tricellular corners [29]. Furthermore, TJ proteins are connected by strands that are dynamic and continuously remodel, break, and regenerate in response to different physiological signals [30,31]. Also, signals such as inflammation modulate the TJ permeability; inflammatory cytokines such as TNF-α and interferon-γ can reorganize the proteins by upregulating the pore-forming claudins while downregulating the sealing claudins, which is studied in patients with inflammatory bowel disease [32,33], meaning that the TJ-mediated permeability is not static but rather dynamic and could be regulated by, for instance, microbiota, inflammation, bile salts, diet, etc.

Artificial modulations of the TJ have been explored in numerous studies as a strategy to enhance oral peptide delivery, which induces mild, transient, and reversible epithelial changes [34]. One notable example is the phase 2 clinical trial of oral insulin 338 (I338), which was co-formulated with the permeation enhancer sodium caprate to open the TJ and facilitate paracellular transport transiently. The study demonstrated that oral dosing could achieve glycemic control comparable to subcutaneous insulin glargine, but only at doses approximately 50–60 times higher, reflecting an oral bioavailability of just 1–2% despite the use of an absorption enhancer [35]. In a Ussing chamber study across ex vivo rat colonic mucosae, human insulin (MW 5.8 kDa [36,37]) marked with fluorescein isothiocyanate (FITC-labeled human insulin) exhibited P_app_ of 0.4 × 10^−6^ cm·s^−1^ [38]. However, co-administration with the permeation enhancer Labrasol^®^ increased insulin permeability 5- to 6-fold ex vivo. In a separate study, the related lipid-based excipient Labrafac^®^ also enhanced intestinal permeability. This shows that permeability and bioavailability of insulin in rats have been significantly increased by the addition of permeation enhancers Labrasol^®^ and Labrafac^®^ [38,39]. Octreotide, an octapeptide somatostatin analog (MW 1.0 kDa), exhibited P_app_ of 3.8 ± 0.3 × 10^−6^ cm·s^−1^ across ex vivo rat colonic mucosa, which was approximately a 2.5-fold increase in the presence of permeation enhancers sodium glycodeoxycholate and sodium deoxycholate [40]. Oral administration of 32-amino acid polypeptide salmon calcitonin (25 mg, MW 3.4 kDa) to fasted beagle dogs resulted in oral bioavailability of 0.3%. Meanwhile, in the presence of citric acid and permeation enhancers lauroyl carnitine chloride and sodium taurodeoxycholate, the bioavailability increased to 1.1 and 1.3%, respectively [41]. Oral administration of exenatide (39-amino acid GLP-1 analog, MW 4.2 kDa) solution to fasted rats did not show any quantifiable bioavailability, while incorporating exenatide into self-nanoemulsifying drug delivery systems increased this parameter to 0.4–0.8% [42].

Altogether, the literature consistently demonstrates that TJ are dynamic structures that regulate paracellular permeability and are modifiable through physiological signaling pathways, inflammatory mediators or permeation enhancers. Much of the evidence linking TJ modulation to peptide transport originates from inflammatory disease models or pharmacological interventions (permeation enhancers) designed to transiently disrupt barrier function. Although these studies provide compelling evidence that increased paracellular permeability can enhance peptide absorption, they do not demonstrate that physiological TJ regulation alone contributes substantially to the natural systemic uptake of intact therapeutic peptides under healthy conditions. This represents an important limitation of the current evidence and highlights the need for further studies investigating peptide transport across intestinal epithelium under physiological conditions, as the TJ strand remodels under physiological conditions as well.

### 2.1. Enzymatic Degradation and Effects of Gastrointestinal pH

Orally administered peptides are exposed to two major obstacles in the gastrointestinal lumen: dynamic pH throughout the GIT and a cascade of proteolytic enzymes, such as pepsin in the stomach and trypsin, chymotrypsin, elastase, carboxypeptidases, and brush-border amino/dipeptidases in the small intestine (Figure 1). In the stomach, the acidic pH (approx. 1.5–3.5) enables the conversion of pepsinogen to pepsin, which preferably cleaves peptide bonds (The peptide bond, –C(=O)–N(H)–, is the result of a reaction between the amino group of one amino acid and the carboxyl group of another amino acid, accompanied by the release of one molecule of water). In contrast, the more neutral pH (approx. 5.5–8.0) of the small intestine promotes the activity of pancreatic proteases, which collectively recognize a broader range of peptide cleavage sites than pepsin [10,25,43]. For instance, pepsin becomes inactive in environments with pH > 5, and trypsin/chymotrypsin work best at higher pH [10]. GIT enzymes cleave peptide bonds at different rates across regions, reducing the fraction of intact therapeutic peptides available to interact with epithelial cells. Partial digestion can produce small fragments of di- or tripeptides that may serve as substrates for intestinal uptake or be small enough to pass through paracellular pores. However, most peptides are either fully degraded or become inactive. Study of the kinetics of degradation and the resultant fragment profile is therefore central to explaining the natural oral bioavailability of peptides. Luminal pH is shown to modulate the processes both by regulating peptide stability and enzyme activity. Also, physiological factors such as food intake, gastric emptying and buffering capacity can alter pH conditions and thereby affect peptide survival.

The enzymatic impact is clearly highlighted in a study that compared the gastrointestinal stability of 17 peptide drugs. The large peptides (including insulin, calcitonin, glucagon, secretin, somatostatin; MW 3.6–5.8 kDa) and containing more than 12 amino acids were degraded within 10 min in natural human gastric fluid containing pepsin (pH 1.8, obtained with the Loc-I-Gut^®^ procedure), whereas the smaller peptides with fewer than 12 amino acids remained stable in the same fluid (Table 1). On the other hand, more rapid degradation (2–10 min) for both large and some of the smaller peptides was observed in natural human small intestinal fluids (obtained with the Loc-I-Gut^®^ procedure from upper jejunum, pH 7.6) containing pancreatic enzymes. The study also compared the same peptides in stimulated gastric (prepared according to USP specifications, pH 1.2, components: NaCl, HCl, pepsin) and intestinal (small intestine, USP specifications pH 6.8, components: NaH_2_PO4, NaOH, pancreatin) fluids without their respective enzymes, in which all peptides showed stability with more than 80% drug recovery within 2 h. Also, protease activity was measured across gastric and intestinal fluids, which demonstrated a strong link between enzyme concentration and peptide degradation rates.

Other experimental studies in simulated gastric (pH 1.2, enzymes: acid-activated porcine pepsin) and intestinal fluids (pH 6.8, enzymes: pancreatin, a mixture of peptidases, amylases, lipases) illustrated the impact of enzymatic degradation and pH effects (Table 1). Degradation half-lives of peptides in these environments vary depending on sequence and structure, ranging from a few minutes for certain endogenous peptides such as somatostatin (13 min in simulated gastric fluid) or oxytocin (8 min), to several hours for more stable analogs such as octreotide or desmopressin (both >24 h) (Table 1) [44]. The same study has revealed that proteolytic digestion typically produces smaller peptide fragments or amino acids, although some cleavage products may retain partial biological activity in some cases. Examples of fragments include: somatostatin ring opening between Phe7-Trp8, oxytocin cleavage between Leu8-Gly9, and linaclotide conversion to desTyr14-linaclotide, which are still pharmacologically active. These peptide properties are underinvestigated. Nevertheless, these results may be attributable to fragmentation representing a loss of intact drug exposure rather than productive absorption [44].

Currently, oral peptide formulation strategies, such as enteric coatings, addition of protease inhibitors, and local pH buffering systems, have been applied. They reduce exposure to degrading enzymes or create environments that temporarily suppress proteolytic activity to increase oral bioavailability. Besides different enteric coatings, citric acid has been co-formulated to lower local small intestinal pH and hence reduce tryptic activity [25]. Protease inhibitors, such as aprotinin, leupeptin, and chicken ovomucoid, are being used in formulations [25]. A clinically relevant example is oral semaglutide, in which co-formulation with absorption enhancer SNAC provides the buffering action by locally increasing the gastric pH, protecting semaglutide from enzymatic degradation, as conversion of pepsinogen is inhibited, which promotes epithelial uptake of the peptide. Combining the effects of the formulation enhancer with fasting further increases the oral bioavailability from approximately 0.8% up to 1.4% [10,24,25,46]. Certain peptides with structural features such as backbone cyclization, constrained conformation, disulfide-rich frameworks and specific amino-acid substitution also showed to limit enzyme access to cleavage sites and thereby increased stability in digestive fluids. An example of this would be cyclotides that resisted GIT degradation. However, they also showed that cyclization alone is not enough, and specific d-amino acid substitutions and backbone changes work considerably better [44]. That said, further studies could provide deeper insight.

Collectively, the literature suggests that enzymatic degradation is not uniformly distributed throughout the gastrointestinal tract. While gastric acidity and pepsin initiate degradation of susceptible peptide drugs, comparative studies indicate that many structurally constrained peptides remain relatively stable in gastric fluid but undergo rapid degradation in the small intestine. This likely reflects the broader substrate specificity and higher cumulative proteolytic activity of pancreatic and brush-border enzymes. Furthermore, peptide susceptibility varies considerably according to molecular architecture, with cyclic peptides, disulfide-rich scaffolds and peptides containing d-amino acids generally exhibiting greater resistance to gastrointestinal proteolysis than larger, flexible linear peptides. However, the contribution of gastric and intestinal degradation is likely to vary between peptide classes.

### 2.2. Impact of Active Transportation (Peptide Transporters and Endocytosis)

Peptide transporters (Figure 1) are essential plasma membrane proteins that assist in the uptake of peptides, mainly di- and tripeptides, as well as certain peptidomimetics like β-lactam antibiotics and ACE-I. In the human gastrointestinal tract, there is primarily expression of the proton-coupled peptide transporter 1 (PEPT1, SLC15A1) and peptide transporter 2 (PEPT2, SLC15A2). Further in the same family of peptide transporters (proton-coupled oligopeptide transporter family) [47,48] there are also peptide/histidine transporters 1 and 2 (PhT1 and PhT2), which are involved in the intracellular transport of histidine-containing peptides [47,49]. PEPT1, which serves as a high-capacity-low-affinity transporter, is predominantly expressed in the small intestine, more specifically, in the brush border on the apical membrane of enterocytes. The highest abundance of PEPT1 expression is in the duodenum and jejunum, decreasing towards the ileum [50]. On the contrary, PEPT2, which displays a high affinity/low-capacity profile, has minor expression in the GIT, specifically in the colon [47]. This regional expression pattern supports the idea that any transporter-mediated peptide absorption is most likely to occur in the proximal small intestine, which goes together with the expression of absorptive enterocytes and a favorable pH.

However, physiological expression alone does not ensure functional uptake of larger peptides, as demonstrated in a study where PEPT1-overexpressing CHO-K1 cells were examined. Cyclic peptides, such as octreotide and pasireotide, can weakly inhibit but are not transported by PEPT1. Both compounds reduced Gly-Sar uptake (IC_50_ = 4.2 mM and 0.53 mM, respectively) without accumulating inside cells compared to parental CHO cells. This suggests a non-transported inhibitory binding with larger cyclic peptides, reinforcing the fact that PEPT1 has a substrate specificity for di- and tripeptides, as well as small peptidomimetics, thus imposing size and structural limitations. The study further implies that other mechanisms, such as organic anion-transporting polypeptide (OATP)-mediated uptake or endocytosis, are likely to contribute to the low but measurable oral bioavailability reported for certain therapeutic peptides [51]. As for PEPT2, almost the same principle applies; it typically recognizes small di-and tripeptides (<600 Da) possessing a free N-terminal amine and C-terminal carboxyl group. All high-affinity substrates typically share several structural features, making the passage of larger >600 Da therapeutic peptides difficult [49]. In addition to uptake transporters, peptides can leave the enterocyte through basolateral peptide transporters that mediate both the influx and the efflux of di- and tripeptides. Other efflux transporters in the GIT, primarily ABC transporters, such as P-glycoprotein, MRP2, MRP3 and BCRP, that limit the absorption of orally administered drugs do not usually have an effect on peptide drugs due to their size [47,48,52,53].

PEPT1 and PEPT2 seem unlikely to contribute substantially to the natural oral absorption of intact therapeutic peptides, despite their central physiological role in intestinal peptide transport. Both transporters exhibit substrate specificity strictly for dipeptides, tripeptides, and selected peptidomimetics that satisfy the strict structural requirements regarding molecular size, geometry, stereochemistry, and proton-coupled binding. Most therapeutic peptides exceed the structural requirements for productive transport. Additionally, recent experimental studies demonstrate that even therapeutic peptides capable of interacting with PEPT1, such as octreotide and pasireotide, are not necessarily transported across the membrane, meaning that transporter binding does not equate to substrate transport. These findings suggest that PEPT1 and PEPT2 primarily function as nutrient transporters optimized for efficient absorption of digestion-derived peptides. Based on this evidence, PEPT1 and PEPT2 are not considered major contributors to the natural oral absorption of intact peptide drugs. An important aspect for future studies could be to determine whether gastrointestinal digestion of therapeutic peptides generates di- or tripeptide fragments that become substrates for PEPT1 and PEPT2.

Endocytosis is another possible mechanism to explain some of the natural oral peptide absorption by cell-specific and transcytosis pathways through epithelial cell types, such as enterocytes, M-cells, goblet cells, and sampling dendritic cells [25]. For example, enterocytes exhibit various endocytic routes, including clathrin-mediated and caveolae-mediated uptake, and surface glycoconjugates like heparan sulfate proteoglycans that can bind and internalize peptides. However, the process of endocytosis also faces practical limitations in the context of oral peptide delivery. Before peptides can interact with the apical membrane and undergo endocytosis, they are exposed to enzymatic degradation, reducing the amount of intact peptide available for cellular uptake. M-cells, on the other hand, contain relatively few lysosomes and can transport intact macromolecules across the epithelium while avoiding digestion, indicating that if the peptide drugs got to the M-cells, the chances of remaining intact and reaching the subepithelial tissue, lymphatic or systemic circulation would be higher. A limiting factor, however, is that M-cells are rare and only make up a small portion of the intestinal surface [25].

Receptor-mediated endocytosis and transcytosis are additional transcellular pathways by which macromolecules may cross the epithelial membrane. Two examples are the neonatal Fc receptor (FcRn) and the transferrin receptor (TfR), which are physiologically expressed in intestinal epithelial cells and have been experimentally investigated for the oral delivery of peptides and proteins. FcRn is expressed in polarized intestinal epithelial cells and mediates the transport of its natural ligands, IgG and albumin. This occurs through a pH-dependent mechanism involving ligand binding, endosomal trafficking, and subsequent basolateral release into a more neutral environment [54] with a pH of about 7.4 [55]. Ligand binding is favored under acidic conditions with a pH between 5.0 and 6.5 [53]. This has been tested using Fc- or albumin-functionalized nanoparticles, resulting in enhanced transepithelial transport of encapsulated peptide cargo such as insulin and exenatide. Receptor specificity is supported by competition experiments and, notably, by the loss of pharmacological response to orally administered insulin-loaded Fc-targeted nanoparticles in FcRN-deficient mice [53,54]. TfR is another potential route; physiologically, transferrin binds to the receptor and undergoes receptor-mediated endocytosis 55]. TfR-targeted systems have demonstrated increased transport across intestinal epithelial models, including enhanced permeability of transferrin-conjugated insulin and transferrin-fractionated nanoparticles. However, the predominantly basolateral localization of TfR in differentiated enterocytes may restrict its accessibility from the intestinal lumen [55,56,57].

Collectively, these findings demonstrate that endogenous receptor-mediated trafficking systems can provide routes for transcellular transport across the intestinal epithelium. However, more evidence derives from engineered receptor-targeted constructs rather than unmodified peptide drugs, so the contribution to the low natural oral absorption of therapeutic peptides remains uncertain.

### 2.3. Microbiota Effect on Barrier Permeability and Absorption

Throughout the gastrointestinal tract, the gut microbiota is expressed in different quantities within the luminal contents, the densest expression being in the distal GIT, i.e., the colon. The gastric and duodenal fluids harbor around 10^1^–10^3^ CFU/mL, increasing towards the ileum (approx. 10^7^ CFU/mL) and reaching its peak in the colonic lumen (10^11^–10^12^ CFU/mL), with a 4-fold increase compared to the proximal parts. This indicates that the highest microbial enzymatic activity happens in the colon, since the stomach and proximal small intestine are too acidic or too aerobic for dense microbial colonization [56]. Some species of gut microbiota, such as *Faecalibacterium prausnitzii* and *Roseburia intestinalis*, and other butyrate-producing bacteria are reported to reduce local inflammation, which improves barrier integrity by tightening the barrier through TJ reassembly but also through other mechanisms such as mucin synthesis or occluding and Zonula occludens-1 protein (ZO-1) upregulation (Figure 1) [57]. This can be attributed to acetate and propionate produced by gut microbiota as well. Other gut microbiota, such as *Akkermansia muciniphila*, as well as other mucus-degrading or mucus-producing bacteria, are noted to enhance mucin production and, respectively, the barrier integrity [57]. While improving TJ integrity decreases paracellular permeability, the bacterial lipopolysaccharide, produced by the Gram-negative bacteria, disrupts the TJ and increases transepithelial permeability. This mechanism is involved in low-grade inflammation seen in conditions like type 2 diabetes and other inflammatory states [57]. Other species, such as *Bacteroides caccae*, which are normally present in the colon, in cases of overgrowth increase mucus degradation by actively interacting and remodeling mucin-type O-glycans in the mucus. That, in turn, causes thinning of the mucosal barrier, contributing to a leaky gut [58]. *Bacteroides fragilis* produces fragilysin (BFT), which cleaves E-cadherin in epithelial cells, disrupting the TJ and triggering inflammation [58]. These bacteria also act as major polysaccharide degraders, providing short-chain fatty acids, vitamins, and metabolites that modulate intestinal barrier function and immune signaling. The same species, through their polysaccharide utilization loci, secrete glycosidases, proteases, and hydrolases via outer membrane vesicles to access complex nutrients, which could be used to degrade macromolecules such as proteins and peptides.

The influence of gut microbiota on intestinal barrier homeostasis is further supported by studies on inflammatory bowel disease, including Crohn’s disease and ulcerative colitis, where dysbiosis is associated with altered mucus integrity and epithelial permeability. Also, microbiota-mucus interaction contributes to mucus barrier homeostasis. In a large cohort study, which was divided into a discovery cohort with 2472 participants and a validation cohort with 655 participants, altered microbial composition and function were associated with impaired intestinal permeability. However, disease-associated alterations in barrier integrity cannot directly be generalized to healthy individuals [59,60].

The available evidence suggests that the microbiota primarily regulates the intestinal environment rather than acting as a major direct determinant of therapeutic peptide bioavailability, although the quantitative contribution of microbial-derived metabolites to endogenous peptide absorption remains poorly understood. Also, dysbiosis-associated inflammation may increase epithelial permeability by disrupting barrier integrity; however, these observations largely stem from pathological conditions and should not be interpreted as evidence that microbial dysbiosis constitutes a physiological mechanism facilitating oral peptide absorption.

### 2.4. Impact of Inflammation-Related Increase in Epithelium Leakiness

The inflammatory cytokines, such as TNF-α, interferon-γ, and IL-13, decrease intestinal epithelial resistance by inducing apoptosis-related changes in TJ (Figure 1, Table 2) [61]. That includes the increased expression of claudin-2 and loss of occludin and claudin-8. In colonic biopsies from Crohn’s disease patients, epithelial resistance decreased by approximately 40%, the TJ strand count decreased by 35%, and epithelial apoptosis increased 2.8-fold [61]. In ulcerative colitis patients, even more severe barrier disruptions were observed, mainly caused by IL-13-induced epithelial damage [61]. Cytokine-specific changes were reported. For instance, TNF-α and interferon-γ were responsible for downregulation of occludins, claudin-5 and -8, while upregulating claudin-2, which is pore-forming. This shifts the TJ toward a cation-leaky state [61,62,63]. IL-13, on the other hand, amplifies leakiness by promoting epithelial apoptosis and microerosion formation in early ulcerative colitis [61]. Inflammatory cytokines also facilitate bacterial and antigen translocation via enhanced endocytosis [61,62,63]. Inflammation contributes to both paracellular and transcellular barrier loss, and although these results are observed in pathological extremes, the same cytokine-driven mechanisms may account for physiological permeability fluctuations relevant to peptide drug permeation.

In a controlled manner, immune activation maintains homeostasis and works to protect barrier integrity; however, excessive cytokine and reactive oxygen species production causes barrier dysfunction. The TJ proteins such as ZO-1 and claudin-4 are also disrupted by inflammatory cytokines, oxidative stress, and apoptosis, leading to increased paracellular permeability in Caco-2 and HT-29 models through NF-kB, MAPK, and MLCK pathways [64]. IL-8 attracts neutrophils, which exacerbate oxidative stress. All of this decreases the TEER while increasing the permeability of experimental permeability markers (e.g., FITC-dextran) [64]. Nonetheless, these changes are reversible, and different agents can be used to restore membrane integrity. For example, TNF-α antibodies or 5-ASA treatment normalize TEER within weeks; probiotics could also help, as microbiota-derived butyrate improves barrier integrity via inhibition of the NF-kB pathway [64]. As discussed previously, dysbiosis of the microbiota, on the other hand, can trigger inflammation (Figure 1) [64].

The sources interpreted in this section demonstrate that GIT inflammation consistently increases epithelial permeability through coordinated alterations in TJ organization, cytokine signaling, apoptosis, etc. However, the vast majority of supporting evidence comes from inflammatory bowel disease or experimentally induced cytokine exposure rather than healthy physiological conditions. Consequently, the enhanced permeability observed during inflammation should be interpreted as a pathological consequence of epithelial barrier dysfunction rather than an endogenous mechanism enabling normal peptide absorption. Although these disease models provide valuable mechanistic insight into the molecular regulation of intestinal permeability, they should not be generalized to healthy individuals or regarded as therapeutically desirable states. The principal value lies in identifying regulatory pathways that can be modulated safely to improve oral peptide delivery without compromising intestinal barrier integrity.

### 2.5. GIT Motility Effect

Gastrointestinal motility determines how long peptides remain in contact with the absorptive epithelium, and several physiological factors influence the residence time. It is characterized by distinct fasting and postprandial motor patterns, quantified by scintigraphy, manometry, and cutaneous electrogastrography. In the fasting state, small intestinal motility is dominated by the migrating motor complex (MMC), which consists of 3 phases. The first phase (40–60 min) is one of prolonged quiescence, phase II (15–40 min) is considered irregular phasic pressure activity, and phase III (15–20 min) consists of rhythmic contractions occurring at approximately 3 cycles/min in the antrum and 11–12 cycles/min in the proximal small bowel propagated over 2–15 min. The forceful contractions of phase III serve as a clearance mechanism, thereby limiting the exposure time of luminal contents to the mucosa. In the fed state, on the other hand, after a mixed meal of ≥400 kcal, the MMC is replaced by a sustained fed pattern that does not have phase III for at least 2 h, resulting in the prolonged intraluminal residence [69].

Additionally, the importance of this hypothesis is underscored by the fact that mucoadhesive polymeric systems, such as polycarbophil-, poly(acrylic acid)-based polymers, cellulose derivatives (e.g., hydroxypropyl cellulose and carboxymethyl cellulose), chitosan, thiolated polymers, the gastrointestinal mucoadhesive patch systems, are currently used in formulations with peptide drugs to extend close contact with the mucosa at the site of drug absorption, thereby preventing the metabolism of peptides *en route* to the absorption sites [70]. The residence time at the site of drug absorption is being increased with the use of these systems, which could achieve site-specific drug delivery and, in turn, increase oral bioavailability of peptide drugs [70]. Poly(acrylic acid)-based polymers, a type of mucoadhesive polymeric system, have been shown to inhibit insulin luminal degradation by trypsin and chymotrypsin.

The pharmacokinetic and pharmacodynamic properties of oral salmon calcitonin formulation with 5-CNAC (8-(N-2-hydroxy-5-chloro-benzoyl)-amino-caprylic acid) in healthy postmenopausal women were markedly affected by water volume and the timing of meal intake [71]. For instance, calcitonin administration with 50 mL of water produced a two- to three-fold increase in plasma C_max_ and AUC compared with 200 mL; time to peak concentration remained rapid (approx. 15 min) and unaffected, suggesting early absorption following gastric emptying [71], meaning that delaying food intake by up to an hour after dosing would enhance bioavailability relative to dosing closer to mealtime. The authors proposed that the acceleration of gastric emptying promotes more rapid delivery of calcitonin to the small intestine, which will reduce exposure to degrading enzymes [71]; however, this finding contradicts the previous one [71].

Quantitative motility measurements demonstrate that small intestinal clearance velocities can vary across physiological and pathological states; for instance, in healthy individuals, phase III contractions propagate through the duodenum–jejunum at speeds ranging from 2.4 to 10 cm/min and persist for approximately 3.6–5.7 min, which indicates rapid clearance of luminal content. However, in patients with Crohn’s disease, marked dysregulation of MMC activity is exhibited, including speeds reaching up to 22.5 cm/min and a complete absence of phase III contractions in approximately 30% of cases. Also, phase II mixing activity gets reduced, and an increase in frequency of propagated contractions is noted, which produces heterogeneous luminal exposure profiles characterized by both rapid displacement and localized peptide pooling. GIT motility strongly modulates spatial and temporal availability of peptides at the epithelial surface, contributing to their possibility to be absorbed [72].

The effect of motility can also be indirectly shown by strong food effects on systemic exposure. Oral salmon calcitonin was studied in both fasting and fed states, where fasting administration produced the highest plasma exposure, with an AUC_0–24h_ of 2431 pg × min × mL^−1^. In contrast, meals consumed 1–4 h before dosing reduced the relative bioavailability by 26–35%. Although times to peak concentrations were similar across conditions, postprandial dosing caused earlier declines and increased variability in calcitonin plasma levels. Gastric emptying, luminal dilution, and altered intestinal flow related to food intake limited the duration and intensity of peptide exposure at absorption sites, constraining oral peptide bioavailability [73]. Additionally, an oral ghrelin agonist ARD-07 tested in healthy volunteers showed that fasting administration resulted in about twice the systemic exposure compared to dosing with food, as indicated by reductions in both AUC_0–24h_ (27.8 ± 4.1 vs. 13.7 ± 1.2) and C_max_ (10.6 ± 1.6 vs. 4.4 ± 0.5 ng × mL^−1^). Despite similar elimination half-lives, food intake delayed and reduced absorption, indicating that luminal factors rather than systemic clearance determine bioavailability [74]. Studies of an oral octreotide formulation (containing polyvinyl pyrrolidone, sodium caprylate, polysorbate 80, glyceryl monocaprylate, glyceryl tricaprylate, and magnesium chloride) in healthy volunteers show that a single 20 mg oral dose produced systemic exposure comparable to a 0.1 mg subcutaneous injection, with similar elimination half-lives (2.38 vs. 2.25 h), and C_max_ (3.77 vs. 3.97 ng/mL) [75]. The time to maximum concentration in the case of oral administration was significantly delayed 2.7 h vs. 0.6 h in subcutaneous administration. However, oral octreotide absorption was highly sensitive to feeding, even in the presence of permeation enhancers. High-fat meals decreased the C_max_ roughly 10-fold, to 0.42 ng/mL, compared to the fasted state, and the therapeutic effect was decreased by approximately 90% [75]. This indicates that native therapeutic peptides, lacking permeation enhancers, are expected to exhibit even lower and more variable absorption under physiological conditions.

Importantly, gastrointestinal motility should not be viewed simply as a determinant of residence time but rather as a dynamic regulator balancing two competing processes, the first one being luminal degradation and the second one, epithelial exposure. Prolonged gastric retention increases peptide exposure to acidic pH and gastric proteases, whereas excessively rapid intestinal transit may shorten the window available for uptake. Optimal oral peptide absorption likely depends on a carefully balanced transit profile rather than uniformly accelerated or delayed gastrointestinal motility. Motility does not seem to work as an independent absorption mechanism but as an indirect physiological regulator. A limitation of current pathological models is that inflammation simultaneously alters several determinants of oral peptide absorption, such as gastric emptying and intestinal motility, and is not solely attributed to epithelial barrier dysfunction or inflammatory signaling. This makes it difficult to distinguish whether changes in systemic peptide exposure result from altered epithelial permeability, modified luminal degradation or differences in gastrointestinal transit. Future studies should investigate whether gastrointestinal motility can be optimized to maximize peptide absorption without altering epithelial barrier integrity, rather than focusing on permeability enhancement; modulation of gastric emptying, intestinal transit and regional drug release may represent more physiological strategies for improving oral peptide drug bioavailability.

## 3. Discussion

Although GIT is evolutionarily adapted to prevent systemic exposure to intact macromolecules, a small but measurable oral absorption of peptide therapeutics has been demonstrated, which suggests that the barriers limiting peptide uptake are not absolute and that physiological processes exist that permit the transport of a small fraction of the administered peptide drug. An observation made across the literature is that the factors explored do not function solely as barriers, but also exhibit a dual role, by simultaneously restricting peptide absorption while also creating opportunities for limited transport. For instance, TJ generally restricts the passage of peptide drugs larger than 400 Da; however, the regional heterogeneity of TJ protein expression, as some of the proteins are considered leakier, could possibly allow for some larger leaks [29]. Also, the strands connecting the proteins are reported to be dynamic rather than static, suggesting that physiological remodeling of TJ proteins could potentially result in a transient increase in epithelial permeability, which in turn could contribute to the small, but measurable amount of oral peptide drug absorption [30,31]. Additionally, they are affected by pathological states such as inflammation, which means that TJ permeability can be regulated by inflammatory cytokines, which could influence the extent of peptide passage. Inflammation also induces apoptosis-related changes in the epithelium and TJ, as well as causes a rearrangement of the expression and disruption of different TJ proteins by the release of inflammatory cytokines [32,33,61,62,63,64]. This shows both paracellular and transcellular contributions to barrier loss; however, the results were observed in pathological extremes. The same cytokine-driven mechanism may account for physiological permeability fluctuations. As for enzymatic degradation and pH-mediated instability, experimental studies have shown that both proteolytic enzymes and pH create a highly unfavorable environment for peptide drugs in the GIT, contributing to their low oral bioavailability [10,45]. However, further investigation into how some peptides survive or transiently evade these degradative processes without permeation enhancers may help clarify the role of enzymes and pH behind the small fraction of natural oral absorption. Additionally, proteolytic degradation generates a diverse range of peptide fragments (including amino acids and smaller peptides), some of which have been reported to retain partial biological activity [44]. Despite this observation, degradation products remain poorly understood; it is therefore possible that enzymatic cleavage could generate bioactive fragments capable of interacting with the intestinal transport mechanism and being absorbed. The current literature predominantly focuses on the loss of intact peptide following gastrointestinal degradation; however, further investigation of stability, absorption, and biological activity of peptide degradation products may reveal new opportunities for oral peptide drug development.

The available evidence suggests that PEPT1 and PEPT2 are unlikely to account for the absorption of intact therapeutic peptides since they exhibit a strong preference for di- and tripeptides as well as small peptidomimetic substrates [49,51]. The molecular weights of therapeutic peptides exceed the substrate range of these transporters, indicating that transport-mediated uptake is unlikely to represent the primary mechanism responsible for natural oral peptide absorption. However, the role should not be completely dismissed, as gastrointestinal degradation generates a range of smaller peptide fragments that may fall within the substrate specificity of the transporters.

The intestinal microbiota appears to exert both advantageous and disadvantageous effects on oral peptide absorption. On one hand, microbial-derived inflammatory signals may disrupt epithelial barrier integrity, which in turn increases paracellular permeability, but on the other hand, some species reduce local inflammation, which strengthens TJ function and thereby limits peptide permeation [57]. In addition, bacterial proteases contribute to peptide degradation [56]. These observations suggest that microbiota should be viewed as a dynamic regulator of intestinal permeability and peptide stability, not only as a barrier.

Gastrointestinal motility influences oral peptide absorption by determining the contact time of peptides in different regions but remains complex, as opposing effects have been reported [70]. Rapid transit time may reduce epithelial exposure and decrease the likelihood of degradation before absorption [71]. On the contrary, there are conditions that prolong luminal residence, such as fasting-state dosing or optimized meal timing, which have been associated with improved bioavailability and should be taken into consideration [74,75]. These conflicting findings may reflect different rate-limiting processes rather than truly conflicting mechanisms. When epithelial permeation is rate-limiting, prolonged residence time may favor absorption, whereas when enzymatic degradation is rate-limiting, shorter transit may preserve a greater fraction of intact peptide. The overall contribution of motility is likely peptide- and context-dependent and determined by the balance between degradation kinetics and time required for epithelial permeability.

Another aspect is that the regional differences across the gastrointestinal tract suggest that the small intestine, particularly the duodenum and jejunum, may provide the most favorable environment for endogenous peptide absorption. The regional advantage seems to arise from the combined balance between epithelial permeability, absorptive area, peptide stability and transit. On the contrary, microbial density and activity increase markedly toward the distal intestine and colon. However, no gastrointestinal region is universally optimal for every peptide or mechanism, but the available evidence identifies the small intestine as the most probable principal site of endogenous absorption.

Although multiple mechanisms have been explored for their potential contribution to oral peptide absorption, the available evidence suggests that not all factors contribute equally. Transporter-mediated uptake via PEPT1 and PEPT2 appears unlikely to account for the absorption of intact therapeutic peptides. In contrast, dynamic regulation of TJ permeability and inflammation-associated alterations in barrier permeability present more plausible mechanisms for the absorption of larger peptide molecules. Gastrointestinal motility and microbiota composition may further influence absorption indirectly by regulating residence time, degradation and epithelial permeability. Collectively, the findings suggest that the measurable oral bioavailability of peptide drugs is unlikely to arise from a single dominant pathway, but rather from the cumulative contribution of multiple low-efficiency physiological processes.

Despite advances in oral drug delivery, important knowledge gaps remain regarding the physiological mechanisms responsible for the small fraction of peptide drugs that is naturally absorbed, as most research has focused on overcoming gastrointestinal barriers through formulation-based approaches. Recently approved oral peptide formulations provide important clinical evidence for the physiological mechanisms discussed. Oral semaglutide combines SNAC-mediated local pH buffering and protection from pepsin degradation with enhanced epithelial passage, whereas the C8-containing formulation of oral octreotide promotes epithelial permeability, including transient modulation of tight junctions. These examples demonstrate that successful oral peptide delivery depends on simultaneously addressing peptide stability and epithelial permeability rather than overcoming a single physiological barrier. Despite formulation enhancers, systemic bioavailability remains approximately 0.8% under recommended dosing conditions and reaches only approximately 1.4% after prolonged fasting. Together with experimental evidence of measurable natural peptide permeability, these observations suggest that formulation technologies enhance intrinsically low physiological transport while multiple remaining barriers continue to restrict overall absorption [24,39,46,76,77].

Future research (Table 3) should not only focus on that part but also on understanding and exploring endogenous absorptive mechanisms that already permit limited uptake. Clinically, this may facilitate the development of oral formulations with improved bioavailability, which could reduce reliance on injectable administration, improve patient adherence, enhance treatment acceptability and expand the therapeutic utility of peptide drugs.

## 4. Conclusions

In conclusion, the available evidence suggests that the limited oral bioavailability of peptide drugs is not solely determined by gastrointestinal barriers but also by physiological mechanisms that permit low levels of absorption. Enzymatic degradation, pH, motility, epithelial transport systems, microbiota, TJ and inflammatory processes appear to function as both barriers and potential facilitators of peptide uptake. Although the importance of these mechanisms remains incompletely understood, together they provide a framework explaining how the small amount of oral bioavailability can occur despite the barriers. However, the mechanisms discussed in this review are unlikely to contribute equally. For instance, dynamic tight-junction regulation currently represents the most plausible endogenous route for intact peptide absorption, whereas transporter-mediated uptake is likely to contribute minimally due to its substrate specificity. Gastrointestinal motility and microbiota appear to act mainly as indirect modulators of absorption, while enzymatic activity and pH determine the amount and form of peptide available for absorption. A more detailed understanding of these natural processes may support the next-generation oral peptide formulation with improved bioavailability and clinical performance, also determining whether peptide degradation products themselves contribute to measurable systemic absorption.

## Figures and Tables

**Table 1:** Degradation half-lives of peptide drugs in simulated gastric and intestinal fluids.

Peptide Drug	MW, kDa	AA Residues, *n*	SGF (pH 1.2 with Pepsin), min	SIF (pH 6.8 with Pancreatin), min	HGF (pH 1.8), min	HIF (pH 7.6), min	PGF (pH 3.4 ± 0.4), min	PIF (pH 6.6 ± 0.2), min	References
Somatostatin º	1.6	14	13 ± 2 [44]; 49 [45]	<3	58	N/A **	98	2.4	[44,45]
Octreotide º	1.0	8	>1440 [44]; 761 [45]	498 ± 60	1071	15	N/A *	554	[44,45]
Oxytocin º	1.0	9	>1440 [44]; N/A * [45]	8 ± 1	405	6.8	884	8.9	[44,45]
Cabetocin º	1.0	8	>1440	13 ± 1	N/A	N/A	N/A	N/A	[44]
Linaclotide	1.5	14	>1440	54 ± 3	N/A	N/A	N/A	N/A	[44]
Plecanatide	1.7	16	>1440	5 ± 1	N/A	N/A	N/A	N/A	[44]
Dolcanatide	1.7	16	>1440	>1440	N/A	N/A	N/A	N/A	[44]
Vasopressin º	1.0	9	>1440	<3.0	N/A	N/A	N/A	N/A	[44]
Desmopressin º	1.1	9	>1440 [44]; N/A * [45]	168 ± 12	633	15	N/A *	63	[44,45]
Nafarelin	1.3	10	357	N/A	491	N/A **	822	14	[45]
Buserelin	1.3	9	324	N/A	424	N/A **	274	1.4	[45]
Goserelin	1.3	10	322	N/A	385	N/A **	376	0.9	[45]
Histrelin	1.3	9	661	N/A	363	1.1	440	3.2	[45]
Leuprolide	1.2	9	166	N/A	267	N/A **	300	1	[45]
[D-Ser]^4^-Gonadorelin	1.2	10	80	N/A	N/A **	N/A **	46	8	[45]
Deslorelin	1.3	9	195	N/A	294	N/A **	468	2.9	[45]
[Arg^8^]-Vasopressin	N/A	9	1093	N/A	468	N/A **	N/A *	2.9	[45]
Ciclosporin º	1.2	11	480	N/A	1374	N/A *	667	N/A *	[45]
Calcitonin	3.4	32	2.1	N/A	2	N/A **	4.6	N/A **	[45]
Glucagon	3.5	29	1.2	N/A	N/A **	N/A **	N/A **	N/A **	[45]
Secretin	3.0	27	N/A **	N/A	N/A **	N/A **	1.1	N/A **	[45]
Insulin	5.8	51	N/A **	N/A	N/A **	N/A **	N/A **	N/A **	[45]

AA—amino acid, SGF—Simulated gastric fluid (USP specifications), SIF—Simulated intestinal fluid (USP specifications), HGF—human gastric fluid, HIF—human intestinal fluid, PGF—pig gastric fluid, PIF—pig intestinal fluid; N/A—data not provided/not assessed, *—degradation half-life not assessed, the peptide is stable, >95% peptide recovery after 2 h (the duration of the experiment), **—degradation half-life was not calculated due to rapid degradation, º—cyclic peptide.

**Table 2:** Effects of inflammatory cytokines on intestinal tight junction proteins. The table summarizes the reported effects of inflammatory cytokines on the expression and organization of tight junction (TJ) proteins involved in maintaining intestinal barrier integrity.

Inflammatory Cytokine	TJ Protein Affected	Change in Expression	References
Tumor Necrosis Factor-α (TNF-α)	Claudin-2 Claudin-1&4 Occludin ZO-1	Upregulated Redistribution from TJ Decreased expression Decreased expression	[64,65,66,67,68]
Interferon-γ	ZO-1 Occludin Claudins	Decreased expression Internalization Upregulation	[64,65,66]
Interleukin-13	Claudin-2	Upregulated	[64]
Interferon-γ + TNF-α	Occludin, Claudin-1&4 JAM-1	Redistribution from TJ Internalization	[64,65]

JAM—junctional adhesion molecules, ZO-1—Zonula Occludens-1 protein.

**Table 3:** Summary table of factors contributing to GIT peptide absorption.

Factor	Supporting Evidence	Limitations	Contribution (Subjective Assessment)	Future Research Needs	References
Enzymatic and pH effects	Experimental and comparative studies demonstrate that gastric and mostly intestinal proteases are major determinants of peptide survival. Peptide stability varies between peptide classes.	Studies focus on degradation rather than measuring absorption. Limited evidence on what happens with the degradation products.	High as it works as an indirect barrier, it is a strong determinant of whether intact peptides remain available for absorption. Although not an absorptive pathway	Quantify the relative contribution of individual gastrointestinal proteases in vivo, investigate whether digestion-derived peptide fragments contribute to systemic exposure via endogenous transport mechanisms	[10,24,25,44,45,46,68]
Tight junctions	The evidence suggests that TJs regulate paracellular permeability. Experimental barrier opening consistently increases peptide permeability.	Most supporting evidence originates from inflammatory disease or permeation enhancer studies rather than healthy physiology.	Moderate	Determine whether physiological TJ dynamic states alone permit systemic uptake	[18,26,27,28,29,30,31,32,33,34,35,36,37,38,39,40,41,42]
Gastrointestinal motility	Fasting, gastric emptying, meal timing and intestinal transit seem to influence oral peptide exposure	Affects the absorption environment rather than directly transporting peptides.	Moderate–low	Investigation of optimal transit profiles and regional delivery strategies without altering epithelial barrier integrity.	[70,71,72,73,74,75]
Microbiota	Seems like microbial metabolites regulate epithelial homeostasis, mucus production and TJ integrity.	Very limited direct evidence on microbiota interaction with therapeutic peptides.	Low	Quantify how microbial-derived metabolites influence peptide absorption under physiological and pathological conditions.	[56,57,58]
Peptide transporters, endocytosis and receptor-mediated endocytosis.	Peptide transporters transport di- and tripeptides and selected peptidomimetics. As for receptor-mediated endocytosis, endogenous intestinal FcRn and TfR can mediate transcellular transport and receptor-targeted insulin, hgH, and nanocarriers show enhanced epithelial uptake/transcytosis. However, the contribution may be limited by ligand specificity as these receptors physiologically recognize specific macromolecules rather than therapeutic peptides.	Exclude intact therapeutic peptides because of size and structural constraints Evidence about FcRN and TfR predominantly derives from engineered fusion proteins or receptor-targeted nanocarriers rather than naturally unmodified therapeutic peptides.	Very low for intact therapeutic peptides, but high for digestion-derived di- and tripeptides Low	Determine whether gastrointestinal degradation generates transportable peptide fragments that contribute to systemic exposure and identify structural features permitting transporter-mediated uptake Determine whether unmodified therapeutic peptides interact with endogenous intestinal receptors at physiologically relevant concentrations.	[47,48,49,51,52,53,54,55,78,79,80]
Inflammation	Strong evidence that inflammatory cytokines increase epithelial permeability through, for instance, TJ remodeling and epithelial injury.	Pathological conditions which should not be generalized to healthy physiology	Negligible under physiological conditions but high under pathological conditions.	Identify physiological regulatory pathways that can safely be modulated without inducing inflammation.	[61,62,63,64,65,66,67,68]

## Data Availability

No new data were created or analyzed in this study. Data sharing is not applicable to this article.

## References

[1] Garvey W.T., Batterham R.L., Bhatta M., Buscemi S., Christensen L.N., Frias J.P., Jódar E., Kandler K., Rigas G., Wadden T.A. (2022). Two-year effects of semaglutide in adults with overweight or obesity: The STEP 5 trial. Nat. Med..

[2] Rahman M.S., Hossain K.S., Das S., Kundu S., Adegoke E.O., Rahman M.A., Hannan M.A., Uddin M.J., Pang M.-G. (2021). Role of Insulin in Health and Disease: An Update. Int. J. Mol. Sci..

[3] Al Shaer D., Al Musaimi O., Albericio F., De La Torre B.G. (2022). 2021 FDA TIDES (Peptides and Oligonucleotides) Harvest. Pharmaceuticals.

[4] Edwards C.M.B., Cohen M.A., Bloom S.R. (1999). Peptides as drugs. QJM.

[5] Nicze M., Borówka M., Dec A., Niemiec A., Bułdak Ł., Okopień B. (2024). The Current and Promising Oral Delivery Methods for Protein- and Peptide-Based Drugs. Int. J. Mol. Sci..

[6] McCartney F., Gleeson J.P., Brayden D.J. (2016). Safety concerns over the use of intestinal permeation enhancers: A mini-review. Tissue Barriers.

[7] Tyagi P., Pechenov S., Anand Subramony J. (2018). Oral peptide delivery: Translational challenges due to physiological effects. J. Control. Release.

[8] Renukuntla J., Vadlapudi A.D., Patel A., Boddu S.H., Mitra A.K. (2013). Approaches for enhancing oral bioavailability of peptides and proteins. Int. J. Pharm..

[9] Brayden D.J., Hill T.A., Fairlie D.P., Maher S., Mrsny R.J. (2020). Systemic delivery of peptides by the oral route: Formulation and medicinal chemistry approaches. Adv. Drug Deliv. Rev..

[10] Baral K.C., Choi K.Y. (2025). Barriers and Strategies for Oral Peptide and Protein Therapeutics Delivery: Update on Clinical Advances. Pharmaceutics.

[11] Brayden D.J., Maher S., Bahar B., Walsh E. (2015). Sodium caprate-induced increases in intestinal permeability and epithelial damage are prevented by misoprostol. Eur. J. Pharm. Biopharm..

[12] Burton P.S., Conradi R.A., Hilgers A.R. (1991). (B) Mechanisms of peptide and protein absorption: (2) Transcellular mechanism of peptide and protein absorption: Passive aspects. Adv. Drug Deliv. Rev..

[13] Nellans H.N. (1991). (B) Mechanisms of peptide and protein absorption: (1) Paracellular intestinal transport: Modulation of absorption. Adv. Drug Deliv. Rev..

[14] Khalid N.-U.-A., Rivera-Delgado E., Von Erlach T. (2026). Navigating the complexity of oral peptide delivery: Challenges and strategies to enhance oral bioavailability. Front. Drug Deliv..

[15] Nielsen D.S., Shepherd N.E., Xu W., Lucke A.J., Stoermer M.J., Fairlie D.P. (2017). Orally Absorbed Cyclic Peptides. Chem. Rev..

[16] Wang C.K., Craik D.J. (2016). Cyclic peptide oral bioavailability: Lessons from the past. Biopolymers.

[17] Panse N., Gerk P.M. (2022). The Caco-2 Model: Modifications and enhancements to improve efficiency and predictive performance. Int. J. Pharm..

[18] Maher S., Brayden D.J. (2012). Overcoming poor permeability: Translating permeation enhancers for oral peptide delivery. Drug. Discov. Today. Technol..

[19] Twarog C., Fattal E., Noiray M., Illel B., Brayden D.J., Taverna M., Hillaireau H. (2022). Characterization of the physicochemical interactions between exenatide and two intestinal permeation enhancers: Sodium caprate (C(10)) and salcaprozate sodium (SNAC). Int. J. Pharm..

[20] Granhall C., Donsmark M., Blicher T.M., Golor G., Søndergaard F.L., Thomsen M., Bækdal T.A. (2019). Safety and Pharmacokinetics of Single and Multiple Ascending Doses of the Novel Oral Human GLP-1 Analogue, Oral Semaglutide, in Healthy Subjects and Subjects with Type 2 Diabetes. Clin. Pharmacokinet..

[21] U.S. Food & Drug Administration Drugs@FDA: FDA-Approved Drugs—Rybelsus. https://www.accessdata.fda.gov/scripts/cder/daf/index.cfm?event=overview.process&ApplNo=213051.

[22] U.S. Food & Drug Administration Drugs@FDA: FDA-Approved Drugs—Wegovy. https://www.accessdata.fda.gov/scripts/CDER/daf/index.cfm?event=overview.process&ApplNo=215256.

[23] Kalra S., Das S., Zargar A.H. (2022). A Review of Oral Semaglutide Available Evidence: A New Era of Management of Diabetes with Peptide in a Pill Form. Indian J. Endocrinol. Metab..

[24] Overgaard R.V., Navarria A., Ingwersen S.H., Bækdal T.A., Kildemoes R.J. (2021). Clinical Pharmacokinetics of Oral Semaglutide: Analyses of Data from Clinical Pharmacology Trials. Clin. Pharmacokinet..

[25] Chen G., Kang W., Li W., Chen S., Gao Y. (2022). Oral delivery of protein and peptide drugs: From non-specific formulation approaches to intestinal cell targeting strategies. Theranostics.

[26] Tsukita S., Furuse M., Itoh M. (2001). Multifunctional strands in tight junctions. Nat. Rev. Mol. Cell Biol..

[27] Zihni C., Mills C., Matter K., Balda M.S. (2016). Tight junctions: From simple barriers to multifunctional molecular gates. Nat. Rev. Mol. Cell Biol..

[28] Anderson J.M., Van Itallie C.M. (2009). Physiology and Function of the Tight Junction. Cold Spring Harb. Perspect. Biol..

[29] Capaldo C.T., Powell D.N., Kalman D. (2017). Layered defense: How mucus and tight junctions seal the intestinal barrier. J. Mol. Med..

[30] Van Itallie C.M., Tietgens A.J., Anderson J.M. (2017). Visualizing the dynamic coupling of claudin strands to the actin cytoskeleton through ZO-1. Mol. Biol. Cell.

[31] Capaldo C.T., Nusrat A. (2015). Claudin switching: Physiological plasticity of the Tight Junction. Semin. Cell Dev. Biol..

[32] Heller F., Florian P., Bojarski C., Richter J., Christ M., Hillenbrand B., Mankertz J., Gitter A.H., Bürgel N., Fromm M. (2005). Interleukin-13 Is the Key Effector Th2 Cytokine in Ulcerative Colitis That Affects Epithelial Tight Junctions, Apoptosis, and Cell Restitution. Gastroenterology.

[33] Zeissig S., Burgel N., Gunzel D., Richter J., Mankertz J., Wahnschaffe U., Kroesen A.J., Zeitz M., Fromm M., Schulzke J.D. (2007). Changes in expression and distribution of claudin 2, 5 and 8 lead to discontinuous tight junctions and barrier dysfunction in active Crohn’s disease. Gut.

[34] Maher S., Leonard T.W., Jacobsen J., Brayden D.J. (2009). Safety and efficacy of sodium caprate in promoting oral drug absorption: From in vitro to the clinic. Adv. Drug Deliv. Rev..

[35] Halberg I.B., Lyby K., Wassermann K., Heise T., Zijlstra E., Plum-Mörschel L. (2019). Efficacy and safety of oral basal insulin versus subcutaneous insulin glargine in type 2 diabetes: A randomised, double-blind, phase 2 trial. Lancet Diabetes Endocrinol..

[36] National Center for Biotechnology Information PubChem Compound Summary for CID 118984375, Insulin Human. https://pubchem.ncbi.nlm.nih.gov/compound/Insulin-Human.

[37] Baeshen N.A., Baeshen M.N., Sheikh A., Bora R.S., Ahmed M.M.M., Ramadan H.A.I., Saini K.S., Redwan E.M. (2014). Cell factories for insulin production. Microb. Cell Fact..

[38] McCartney F., Jannin V., Chevrier S., Boulghobra H., Hristov D.R., Ritter N., Miolane C., Chavant Y., Demarne F., Brayden D.J. (2019). Labrasol^®^ is an efficacious intestinal permeation enhancer across rat intestine: Ex vivo and in vivo rat studies. J. Control. Release.

[39] McCartney F., Caisse P., Dumont C., Brayden D.J. (2024). Labrafac™ MC60 is an efficacious intestinal permeation enhancer for macromolecules: Comparisons with Labrasol^®^ ALF in ex vivo and in vivo rat studies. Int. J. Pharm..

[40] Brayden D.J., Stuettgen V. (2021). Sodium glycodeoxycholate and sodium deoxycholate as epithelial permeation enhancers: In vitro and ex vivo intestinal and buccal bioassays. Eur. J. Pharm. Sci..

[41] Sinko P.J., Lee Y.-H., Makhey V., Leesman G.D., Sutyak J.P., Yu H., Perry B., Smith C.L., Hu P., Wagner E.J. (1999). Biopharmaceutical Approaches for Developing and Assessing Oral Peptide Delivery Strategies and Systems: In Vitro Permeability and In Vivo Oral Absorption of Salmon Calcitonin. Pharm. Res..

[42] Venkatasubramanian R., Al-Maghrabi P.M., Alavi O., Lind T., Sassene P.J., Kirkensgaard J.J.K., Mota-Santiago P., Rades T., Müllertz A. (2025). Design, evaluation, and in vitro–in vivo correlation of self-nanoemulsifying drug delivery systems to improve the oral absorption of exenatide. J. Control. Release.

[43] Ma B., Fuhrmann J., Henriksen H., Khojasteh S.C., Li W., Liu J., Plise E., Yu Q., Cheruzel L. (2025). Overcoming Challenges in the Metabolism of Peptide Therapeutics: Strategies and Case Studies for Clinical Success. J. Med. Chem..

[44] Kremsmayr T., Aljnabi A., Blanco-Canosa J.B., Tran H.N.T., Emidio N.B., Muttenthaler M. (2022). On the Utility of Chemical Strategies to Improve Peptide Gut Stability. J. Med. Chem..

[45] Wang J., Yadav V., Smart A.L., Tajiri S., Basit A.W. (2015). Toward Oral Delivery of Biopharmaceuticals: An Assessment of the Gastrointestinal Stability of 17 Peptide Drugs. Mol. Pharm..

[46] Aroda V.R., Blonde L., Pratley R.E. (2022). A new era for oral peptides: SNAC and the development of oral semaglutide for the treatment of type 2 diabetes. Rev. Endocr. Metab. Disord..

[47] Rubio-Aliaga I., Daniel H. (2002). Mammalian peptide transporters as targets for drug delivery. Trends Pharmacol. Sci..

[48] Daniel H. (2004). Molecular and Integrative Physiology of Intestinal Peptide Transport. Annu. Rev. Physiol..

[49] Wang C., Chu C., Ji X., Luo G., Xu C., He H., Yao J., Wu J., Hu J., Jin Y. (2022). Biology of Peptide Transporter 2 in Mammals: New Insights into Its Function, Structure and Regulation. Cells.

[50] Lee V.H.L. (2000). Membrane transporters. Eur. J. Pharm. Sci..

[51] Bajraktari-Sylejmani G., Von Linde T., Burhenne J., Haefeli W.E., Sauter M., Weiss J. (2022). Evaluation of PepT1 (SLC15A1) Substrate Characteristics of Therapeutic Cyclic Peptides. Pharmaceutics.

[52] Murakami T., Takano M. (2008). Intestinal efflux transporters and drug absorption. Expert Opin. Drug Metab. Toxicol..

[53] Wacher V.J., Silverman J.A., Zhang Y., Benet L.Z. (1998). Role of P-Glycoprotein and Cytochrome P450 3A in Limiting Oral Absorption of Peptides and Peptidomimetics. J. Pharm. Sci..

[54] Azevedo C., Nilsen J., Grevys A., Nunes R., Andersen J.T., Sarmento B. (2020). Engineered albumin-functionalized nanoparticles for improved FcRn binding enhance oral delivery of insulin. J. Control. Release.

[55] Pridgen E.M., Alexis F., Kuo T.T., Levy-Nissenbaum E., Karnik R., Blumberg R.S., Langer R., Farokhzad O.C. (2013). Transepithelial Transport of Fc-Targeted Nanoparticles by the Neonatal Fc Receptor for Oral Delivery. Sci. Transl. Med..

[56] McCallum G., Tropini C. (2024). The gut microbiota and its biogeography. Nat. Rev. Microbiol..

[57] Allam-Ndoul B., Castonguay-Paradis S., Veilleux A. (2020). Gut Microbiota and Intestinal Trans-Epithelial Permeability. Int. J. Mol. Sci..

[58] Zafar H., Saier M.H. (2021). Gut *Bacteroides* species in health and disease. Gut Microbes.

[59] Leibovitzh H., Lee S.-H., Xue M., Raygoza Garay J.A., Hernandez-Rocha C., Madsen K.L., Meddings J.B., Guttman D.S., Espin-Garcia O., Smith M.I. (2022). Altered Gut Microbiome Composition and Function Are Associated With Gut Barrier Dysfunction in Healthy Relatives of Patients With Crohn’s Disease. Gastroenterology.

[60] Dunleavy K.A., Raffals L.E., Camilleri M. (2023). Intestinal Barrier Dysfunction in Inflammatory Bowel Disease: Underpinning Pathogenesis and Therapeutics. Dig. Dis. Sci..

[61] Schulzke J.D., Ploeger S., Amasheh M., Fromm A., Zeissig S., Troeger H., Richter J., Bojarski C., Schumann M., Fromm M. (2009). Epithelial Tight Junctions in Intestinal Inflammation. Ann. N. Y. Acad. Sci..

[62] Cui W., Li L.X., Sun C.M., Wen Y., Zhou Y., Dong Y.L., Liu P. (2010). Tumor necrosis factor alpha increases epithelial barrier permeability by disrupting tight junctions in Caco-2 cells. Braz. J. Med. Biol. Res..

[63] Poritz L.S., Harris L.R., Kelly A.A., Koltun W.A. (2011). Increase in the tight junction protein claudin-1 in intestinal inflammation. Dig. Dis. Sci..

[64] John L.J., Fromm M., Schulzke J.-D. (2011). Epithelial Barriers in Intestinal Inflammation. Antioxid. Redox Signal..

[65] Bruewer M., Luegering A., Kucharzik T., Parkos C.A., Madara J.L., Hopkins A.M., Nusrat A. (2003). Proinflammatory Cytokines Disrupt Epithelial Barrier Function by Apoptosis-Independent Mechanisms. J. Immunol..

[66] Capaldo C.T., Nusrat A. (2009). Cytokine regulation of tight junctions. Biochim. Biophys. Acta.

[67] Luettig J., Rosenthal R., Barmeyer C., Schulzke J.D. (2015). Claudin-2 as a mediator of leaky gut barrier during intestinal inflammation. Tissue Barriers.

[68] Ma T.Y., Iwamoto G.K., Hoa N.T., Akotia V., Pedram A., Boivin M.A., Said H.M. (2004). TNF-alpha-induced increase in intestinal epithelial tight junction permeability requires NF-kappa B activation. Am. J. Physiol. Gastrointest. Liver Physiol..

[69] Camilleri M., Hasler W.L., Parkman H.P., Quigley E.M.M., Soffer E. (1998). Measurement of Gastrointestinal Motility in the GI Laboratory. Gastroenterology.

[70] Shaji J., Patole V. (2008). Protein and Peptide Drug Delivery: Oral Approaches. Indian J. Pharm. Sci..

[71] Karsdal M.A., Byrjalsen I., Riis B.J., Christiansen C. (2008). Optimizing bioavailability of oral administration of small peptides through pharmacokinetic and pharmacodynamic parameters: The effect of water and timing of meal intake on oral delivery of Salmon Calcitonin. BMC Clin. Pharmacol..

[72] Annese V., Bassotti G., Napolitano G., Usai P., Andriulli A., Vantrappen G. (1997). Gastrointestinal Motility Disorders in Patients with Inactive Crohn’s Disease. Scand. J. Gastroenterol..

[73] Karsdal M.A., Byrjalsen I., Azria M., Arnold M., Choi L., Riis B.J., Christiansen C. (2009). Influence of food intake on the bioavailability and efficacy of oral calcitonin. Br. J. Clin. Pharmacol..

[74] MacLean C.M., Casanova A.-T., Baselgia-Jeker L., Neave N., Larsen F., Skillern L., Drewe J., Beglinger C. (2009). Effect of Food on the Pharmacokinetics and Pharmacodynamics of an Oral Ghrelin Agonist (ARD-07) in Healthy Subjects. J. Clin. Pharmacol..

[75] Tuvia S., Atsmon J., Teichman S.L., Katz S., Salama P., Pelled D., Landau I., Karmeli I., Bidlingmaier M., Strasburger C.J. (2012). Oral Octreotide Absorption in Human Subjects: Comparable Pharmacokinetics to Parenteral Octreotide and Effective Growth Hormone Suppression. J. Clin. Endocrinol. Metab..

[76] Fattah S., Ismaiel M., Murphy B., Rulikowska A., Frias J.M., Winter D.C., Brayden D.J. (2020). Salcaprozate sodium (SNAC) enhances permeability of octreotide across isolated rat and human intestinal epithelial mucosae in Ussing chambers. Eur. J. Pharm. Sci..

[77] Maher S., Brayden D.J. (2021). Formulation strategies to improve the efficacy of intestinal permeation enhancers. Adv. Drug Deliv. Rev..

[78] Du W., Fan Y., Zheng N., He B., Yuan L., Zhang H., Wang X., Wang J., Zhang X., Zhang Q. (2013). Transferrin receptor specific nanocarriers conjugated with functional 7peptide for oral drug delivery. Biomaterials.

[79] Bobst C.E., Wang S., Shen W.-C., Kaltashov I.A. (2012). Mass spectrometry study of a transferrin-based protein drug reveals the key role of protein aggregation for successful oral delivery. Proc. Natl. Acad. Sci. USA.

[80] Yong J.M., Mantaj J., Cheng Y., Vllasaliu D. (2019). Delivery of Nanoparticles across the Intestinal Epithelium via the Transferrin Transport Pathway. Pharmaceutics.

